# Procerus sign as a diagnostic clue for progressive supranuclear palsy

**DOI:** 10.1055/s-0046-1824436

**Published:** 2026-06-23

**Authors:** Gabriel de Deus Vieira, Ed Cleso Pereira de Souza Filho, Gabriel Erzinger, Miline Weis Becker, Marcelo Henrique de Moura Campos, Eduardo Rosa, Laura Fiuza Parolin, Marcus Vinícius Magno Gonçalves, Ylmar Corrêa Neto

**Affiliations:** 1 Universidade Estadual de Campinas, Faculdade de Ciências Médicas, Laboratório de Neuroimagem, Campinas SP, Brazil.; 2 Universidade da Região de Joinville, Departamento de Medicina, Disciplina de Neurologia, Joinville SC, Brazil.; 3 Universidade Federal de Santa Catarina, Hospital Universitário Professor Polydoro Ernani de São Thiago, Serviço de Neurologia, Florianópolis SC, Brazil

**Keywords:** Supranuclear Palsy, Progressive, Parkinsonism, Signs and Symptoms

## Abstract

The procerus sign is associated with some diseases that initially manifest as atypical parkinsonism, especially progressive supranuclear palsy (PSP). It is described as a vertical contraction of the muscles in the glabellar region. This sign helps to clinically differentiate atypical parkinsonism diseases, especially in the early stages, when many conditions are similar, in terms of clinical presentation and poor response to levodopa. Thus, the procerus sign can be an important clue in the suspicion of PSP.

## INTRODUCTION

The procerus sign (PS) was first described by Roman and Colosimo in 2001 as a 'worried' expression. 1 However, in 1872, it is believed that Charles Darwin was already aware of this sign, calling it the *Omega sign* , which was used to describe melancholy in patients with severe psychomotor disorders. 2 3 The word procerus is derived from Latin, meaning tall or long, and is a reference to the procerus facial muscle, having an important function in the eyebrow movement. 4 5 This sign is related to atypical parkinsonism diseases, especially progressive supranuclear palsy (PSP) and some cases of corticobasal syndrome (CBS). 3 6 Thus, this study aims to review the vertical glabelar winkle, also known as the procerus sign.

## PROGRESSIVE SUPRANUCLEAR PALSY

This is an uncommon neurodegenerative disease, first described in its neuropathology in 1964 by Steele, Richardson, and Olszewski. 7 8 9 It was probably previously described by Jean-Martin Charcot in 1888, when he reported a man with significant rigidity without tremor. 10 Usually, it affects the basal ganglia, the integumentary portion of the brainstem, and the cerebral cortex. 7 Among the subtypes, the most common are Richardson syndrome (PSP-RS) and PSP with predominant Parkinsonism (PSP-P). 11

The prevalence of PSP in its classic form is around 5 to 7 cases per 100 thousand individuals, 8 with patients being on average 60 years old when symptoms begin. 6 Clinically, PSP-RS initially presents with difficulty in walking, balance, cogwheel stiffness, falls from one's own height, paralysis of the vertical gaze, and progressive cognitive decline. Furthermore, PSP-P presents a rigid-hypokinetic form, but with a late onset of gaze paralysis. 8 11 Both forms have a poor response to levodopa. 8 10

## ANATOMICAL DETAILS

**Figure 1:**
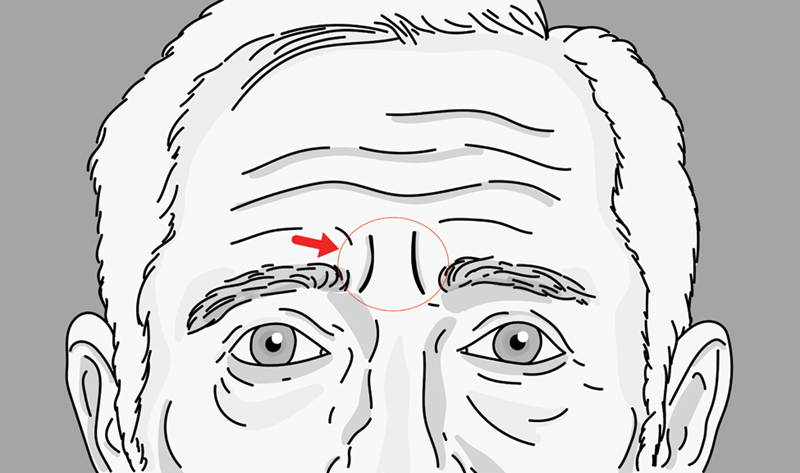
Procerus sign (circle and red arrow).

The procerus sign is a facial dystonia of the muscles in the glabellar region, leading to the formation of vertical wrinkles, which can extend to the dorsum of the nose. 3 It occurs because the procerus muscle contracts the medial angle of the eyebrows downwards (generates transverse wrinkles), the corrugator muscle of the eyebrow contracts the eyebrows medially downwards (generates vertical wrinkles), and the entire complex of the orbicularis oculi muscle responsible for the contraction of the forehead, temple, and cheek becomes responsible for wrinkles towards the medial angle of the orbit ( Figure 1 ). 3 12 Another hypothesis is that this sign arises due to dyskinesia of the procerus muscle and facial bradykinesia secondary to Parkinsonism. 1 13

## IMPACT ON PSP DIAGNOSIS

Clinically, differentiating neurodegenerative parkinsonian syndromes is a challenge in clinical practice, since there are no biological markers for atypical parkinsonism types, with all syndromes being very similar at symptom onset. 2 14 Litvan et al. 15 proposed a diagnostic model that cites PS, adding it as a sign that may be present in PSP, prioritizing sensitivity over specificity. 15 In the most recent criteria, published in 2017, 16 four core manifestation domains were cited: ocular motor dysfunction, postural instability, akinesia, and cognitive dysfunction; however, there is no mention of PS. 16

According to the study by Warabi et al. 13 soon after the first description of this sign in patients with PSP, the authors reported the same finding in patients with CBS. The neuropathological similarities in PSP and CBS, such as the presence of tau haplotype, ocularmotor dysfunction, and PS, as they fulfill criteria for possible 4-repeat haplotype (4R)-tauopathies together, as a recent Brazilian work 17 hypothesized was the presence of this sign in both pathologies. 13 17 18

It is known that PSP has a common pathology with CBS, characterized by the existence of neurofibrillary tangles in the substantia nigra and glial tangles, in addition to tau isoforms of 4R. 19 The tau protein is associated with the formation of microtubules and maintenance of the structural integrity of axons; it has two haploids, H1 and H2, and the H1/H1 genotype, being a risk factor for PSP. 11 19 Thus, the expression of tau 4R cannot resist microtubular dysfunction leading to degeneration. 6 19 Consequently, PS possibly occurs due to degeneration outside the substantia nigra, though only histopathological studies can confirm whether this sign is a generic manifestation of tautopathy or specific to PSP. 3

## CONCLUSION

Currently, the use of the procerus sign for PSP diagnosis criteria remains uncertain. However, this sign is a common finding in PSP, being found in the early stages of the disease. While it is not yet possible to use this sign as a diagnostic criterion for PSP, its close association with the disease helps in the diagnostic suspicion. Further studies on this topic should be encouraged to establish the real prevalence of PS in this cohort and other types of atypical parkinsonism.
